# Laboratory-Simulated Inhibitory Effects of the Floating-Bed Plants on *Microcystis aeruginosa* and Their Microbial Communities’ Responses to Microcystins

**DOI:** 10.3390/microorganisms12102035

**Published:** 2024-10-09

**Authors:** Shuwen Zhang, Yuanpu Sha, Yuanyuan Tang, Longjie Li, Feihu Wang, Jing Dong, Xuejun Li, Yunni Gao, Xiaofei Gao, Huatao Yuan, Jingxiao Zhang

**Affiliations:** 1 College of Fisheries, Henan Normal University, Jianshe Road, Xinxiang 453007, China; fantastic_1219@163.com (S.Z.);; 2 Observation and Research Station on Water Ecosystem in Danjiangkou Reservoir of Henan Province, Nanyang 474450, China

**Keywords:** algal inhibition, antioxidant system, inter-root microorganisms, MCs-degrading bacteria, eutrophic water bodies

## Abstract

Three common floating bed plants, *Eichhornia crassipes*, *Pistia stratiotes*, and *Ipomoea aquatica*, were selected in the present study to investigate their inhibitory effects on toxic *Microcystis aeruginosa*. The results showed that all three types of floating-bed plants could considerably inhibit the growth of *M. aeruginosa* and effectively remove the microcystins (MCs) from water systems, among which, *E. crassipes* and *P. stratiotes* were more effective in resisting *M. aeruginosa*, and the removal rate of the intracellular MCs could be up to 100%. In addition, the roots and leaves of the three plants were enriched with a large number of MCs and demonstrated significant antioxidant responses, as evidenced by the increase in the content of catalase (CAT), glutathione peroxidase (GSH-PX), superoxide dismutase (SOD), and malondialdehyde (MDA) in the roots, stems, and leaves of the plants. Furthermore, this study also showed that Proteobacteria, Bacteroidota, Myxococcota, Verrucomicrobiota, and Actinobacteriota dominated the root microorganisms of the three plants. Moreover, a variety of MC-degrading bacteria, including *Sphingomonas*, *Acinetobacter*, *Novosphingobium*, and *Pseudomonas*, were found at the genus level, which further provides important basic data for the regulation of eutrophic water bodies and the removal of MCs.

## 1. Introduction

In recent years, with the rapid development of industry and economy, aquatic ecosystems have suffered serious threats of pollution and damage [1], and eutrophication-induced cyanobacterial blooms have become a hot topic for aquatic ecosystem studies [2]. It has been shown that the occurring frequency of cyanobacterial blooms might be related to climate change, human activities, and nutrient discharges from land [3]. *Microcystis* is the most common microalga in freshwater ecosystems, where cyanobacterial blooms have occurred in recent years [4]. *M. aeruginosa* is divided into two main types: toxin and non-toxin-producing strains [5]. Toxigenic *M. aeruginosa* can release microcystins (MCs) into the aquatic environment, which can be enriched in aquatic organisms and, thus, threaten human health via the food chain [6].

Currently, various methods have been used to deal with cyanobacterial blooms, of which the biological method is considered to be a long-term, green, and healthy method [7]. In freshwater ecosystems, both aquatic macrophytes and phytoplankton are important producers, and competition between them has been proven to play an important role in maintaining the health and stability of aquatic ecosystems [8]. The interaction between macrophytes and phytoplankton mainly includes competition for light, nutrients, living space, and allelopathy, which is the release of active substances by macrophytes, thereby inhibiting the growth of phytoplankton [9]. Li & Hu (2005) [10] showed that the aquatic plant *Phragmites communis* could inhibit the growth of *M. aeruginosa*. Chen et al. (2012) [11] found that *Nymphaea tetragona*, *Typha orientalis*, *Nelumbo nucifera*, and *Iris wilsonii* demonstrated strong inhibition of *M. aeruginosa*, with inhibition rates of 75–82%.

Although there have been many successful practices for the restoration of cyanobacterial blooms by aquatic macrophytes, their effectiveness is still affected by environmental factors, such as water depth, wind, and waves. In recent years, ecological floating beds (EFB), as an in situ remediation and control technology for eutrophic waters, have been widely practiced because of their advantages of saving energy and space resources [12,13,14]. Based on their biological self-purification ability, plants are among the most important factors determining the purification capacity of floating bed systems, and variations exist in the purification effects of different plants [15,16]. Generally, during the degradation of toxic microcystin-producing cyanobacteria, tens of times more MCs are released into water bodies [17]. Consequently, to assess the feasibility of employing floating bed plants for the restoration of microcystin-contaminated water bodies, the MCs content in the water and the tolerance of the plants to MCs are among the factors that must be considered in this study. Previous research has primarily focused on the effects of MCs on submerged plants. Calado et al. (2019) [18] discovered through in vitro simulation that MC-LR had no significant effect on the antioxidant enzyme activity of *Myriophyllum verticillatum*, *Ceratophyllum demersum*, and *Elodea densa* Casp. Nevertheless, Rojo et al. (2013) [19] found that MC-LR could significantly inhibit the growth of *Myriophyllum verticillatum* and that cyanobacterial bloom extract could induce oxidative stress injury in *Vallisneria natans* [17,20]. The disparity in these experimental results might be associated with the concentration of toxins and the tolerance of the plants. However, there are few reports on the responses of different floating-bed plants to MCs. In addition, the root systems of floating bed plants are more developed and can attach a large number of microorganisms. A microbial membrane is an important form of microbial immobilization that can be used to biodegrade MCs in water bodies [21]. The root systems of floating-bed plants are in direct contact with *M. aeruginosa* during freshwater ecosystem restoration and microalgae suppression. However, to date, the microorganisms associated with floating bed plants and the microbial responses to MCs during water restoration are poorly understood. The microbial communities of plants might play an important role in the removal of MCs and the practical application of microalgae inhibition. Thus, exploring the algal inhibition effects of floating plants on *M. aeruginosa* and the microbial community response to MCs will have important research value and considerable significance for the management and restoration of cyanobacterial blooms in aquatic ecosystems.

*E. crassipes*, *P. stratiotes,* and *I. aquatica* are widely used in ecological floating-bed systems. Previous studies have indicated that allelochemicals secreted by the roots of *E. crassipes* could demonstrate good inhibitory effects on *M*. *aeruginosa* [22]. Wu et al. (2015) [23] also suggested that *P. stratiotes* could significantly inhibit the growth of *M. aeruginosa* [23]. *I. aquatica* has also been shown to have the potential to remediate eutrophic waterbodies [24]. In the present study, *E. crassipes*, *P. stratiotes*, and *I. aquatica* were selected as floating bed plants to simulate the restoration of eutrophic water bodies with toxin-producing *M. aeruginosa* in BG-11 culture medium. This study aimed to provide a theoretical basis for a comprehensive evaluation of the restoration effects of floating-bed plants on cyanobacterial blooms and provide a good choice for the restoration of eutrophic water bodies.

## 2. Materials and Methods

### 2.1. Experimental Material

*M. aeruginosa* (FACHB-905) was purchased from the Freshwater Algae Culture Collection of the Institute of Hydrobiology, Chinese Academy of Sciences. *M. aeruginosa* (FACHB-905), selected for this study, was a toxin-producing strain that could produce and secrete MCs [25]. Before the formal experimentation, *M. aeruginosa* was acclimated in a sterile BG-11 culture medium in a constant-temperature light incubator until the exponential growth phase. The culture conditions were 25 °C with a light intensity of 2500 lx and a photoperiod of 12 h light:12 h dark.

The experimental plants, *E. crassipes* and *P. stratiotes*, were purchased from Ye Cotton Xuan Florist, Luzhi, Wuzhong District, Suzhou City, Jiangsu Province, and *I. aquatica* was purchased from Seed Market, Logistics Park, Wenjia Street, Weifang City, Shandong Province. All purchased plants were cleaned with a soft-bristled brush and hydroponically domesticated for one week before the experiment [26]. In order to avoid the interference of plant shading effects, the control group was also set by using plastic plants, which were purchased from Yiwu Tilly Arts and Crafts Factory, and the plastic grass used was soaked in alcohol for 48 h and then rinsed repeatedly in sterile water for formal experiments.

### 2.2. Experimental Design

**Figure 1:**
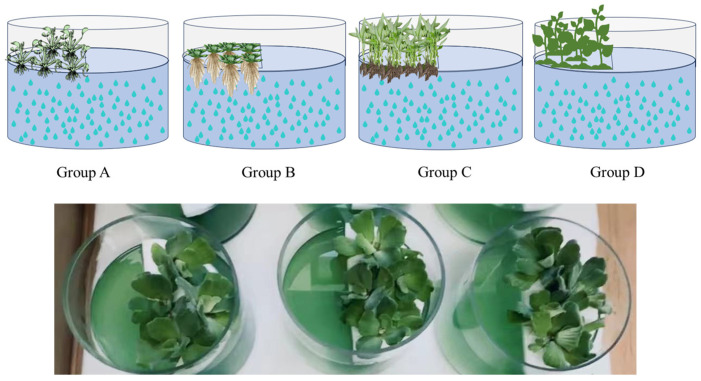
Experimental design of the co-cultivation of floating-bed plant with toxin-producing *M. aeruginosa* (Group A: *M. aeruginosa* with *E. crassipes*; Group B: *M. aeruginosa* with *P. stratiotes*; Group C: *M. aeruginosa* with *I. aquatica*; Group D: *M. aeruginosa* with plastic grass).

The simulation experiment was carried out in a 7 L cylindrical glass tank with 5 L of *M. aeruginosa* (initial optical density at 665 nm, OD_665_ = 0.1, and the initial *M. aeruginosa* density was 0.68 × 10^6^ cells/mL). As shown in Figure 1, the treatment with a floating bed system with 50% coverage of *E. crassipes* was designated as Group A, Group B was *P. stratiotes*, and Group C was *I. aquatica*, while that with plastic grass was designated as the control (Group D). The entire experimental period lasted for 15 days.

### 2.3. Sample Collection and Measurement

Microalgae samples of *M. aeruginosa* were collected on days 0, 2, 4, 6, 9, 12, and 15 for the determination of OD_665_, *M. aeruginosa* density, and chlorophyll a. In addition, at the beginning and end of the experiment, 1 mL of *M. aeruginosa* samples from each group were collected and centrifuged at 8000× *g* for 15 min. Thereafter, 50 μL of the supernatant was collected and used for the determination of extracellular toxins using a microcystin ELISA kit purchased from the Institute of Hydrobiology, Chinese Academy of Sciences. The ELISA kit specifies the conditions and detailed analytical steps for the determination of microcystins (cyclic pentapeptides) in water by high-efficiency liquid chromatography and indirect competitive enzymes combined with immunosorption, applicable to the determination of microcystins in drinking water, lake water, river water, and surface water. The detection limit of microcystins in the samples HPLC and ELISA were both 0.1 μg/L, and suitable microcystin assay types were MC-RR, MC-YR, and MC-LR.

The root, stem, and leaf tissues of each experimental plant were collected on days 0, 7, and 15 to determine the enzyme activities of the antioxidant systems. Plant tissue (0.1 g) was placed in a 2 mL centrifuge tube, and 1 mL of 0.1 mol/L^−1^ phosphate buffer solution (PBS) was added, followed by repeated grinding with a cell grinder (65 Hz, 300 s, 2 times) for effective cell fragmentation. The lysed cells were centrifuged at 8000× *g* for 15 min at 4 °C, and the supernatant was collected for the determination of catalase (CAT), glutathione peroxidase (GSH-PX), superoxide dismutase (SOD), and malondialdehyde (MDA) using a kit purchased from the NanJing JianCheng Bio-engineering Institute, NanJing, China.

In addition, microbial membrane samples of the roots of each plant were collected at the beginning and end of the experiments. The root tissue (1 g) was weighed, placed in sterile centrifuge tubes, washed with 10 mL of 0.1 mol/L^−1^ PBS, and ultrasonicated for 1 min, repeated three times. After three washes, the washing solution was collected and filtered with a 0.22 μm acetate fiber filter membrane. Thereafter, the filter membrane was placed in a 10 mL sterile centrifuge tube and stored at −80 °C for high-throughput sequencing to analyze the composition of the microbial communities. The high-throughput sequencing service was provided by Shanghai Meiji Bio-medical Technology Co., Ltd., Shanghai, China, and community composition analysis was conducted by Meiji Bio online analysis. software (http://www.majorbio.com/). accessed on 21 March 2024.

### 2.4. Data Analysis

The resulting data were statistically analyzed using Microsoft Excel 2019 and SPSS 26.0, while graphical analysis was conducted using Origin Pro 2024 software. One-way ANOVA and *t*-tests were employed to assess the significance of differences in the antioxidant response of the plants and MC-LR content among the various groups. Additionally, a repeated-measures analysis method was used to evaluate the significant differences in the growth of *M. aeruginosa* (FACHB-905). Statistical significance was set at *p* < 0.05. The diversity of microbial communities in plant root biofilms was described and analyzed using the Majorbio I-Sanger Cloud Platform (https://cloud.majorbio.com). accessed on 23 March 2024.

## 3. Results and Analysis

### 3.1. Effects of Different Floating Bed Plants on the Growth of M. aeruginosa in the Control and Each Treatment

**Figure 2:**
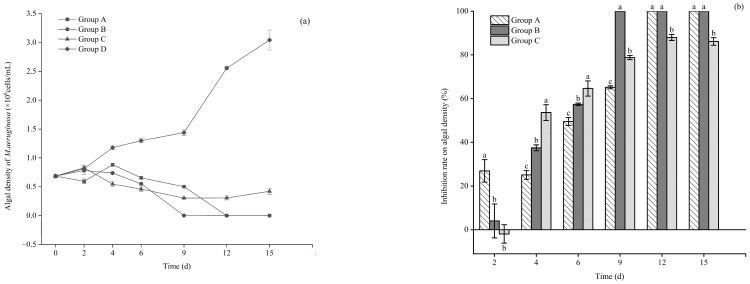
*M. aeruginosa* density changes (**a**) and the inhibition rate (**b**) of *M. aeruginosa* in each treatment group. Notes: The letters indicate the differences between groups on different days, the same letters on the same day indicate that the differences between groups are not significant (*p* > 0.05), and the different letters indicate significant differences (*p* < 0.05).

As shown in Figure 2a, the *M. aeruginosa* density in the control group continued to increase to 3.04 × 10^6^ cells/mL at the end of the experiment; however, the value significantly decreased from day 6 to 0.65 × 10^6^ cells/mL and 0.55 × 10^6^ cells/mL, respectively, with *E. crassipes* or *P. stratiotes*. The inhibition rate of *M. aeruginosa* by *P. stratiotes* or *E. crassipes* was up to 100% on days 9 and 12, respectively (Figure 2b). Meanwhile, the microalgae density of *M. aeruginosa* in the presence of *I. aquatica* also showed a significant decrease from day 4, reducing to 0.54 × 10^6^ cells/mL, but little change was detected from day 6 to day 15. With respect to statistically repeated measurements, it was suggested that significant group effects (F = 60.135, *p* < 0.001), time effects (F = 517.82; *p* < 0.001), and group × time effects (F = 2834.96; *p* < 0.001) were detected in the microalgae density of *M. aeruginosa* during the entire experiment.

### 3.2. Effect of Different Floating Bed Plants on the Chl a (Chlorophyll a) Content of M. aeruginosa

**Figure 3:**
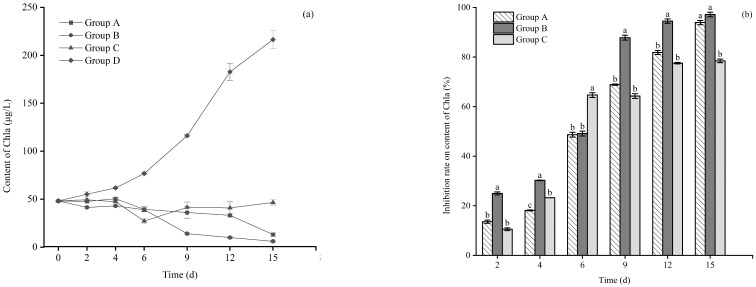
Content of Chl a (**a**) and the inhibition rate (**b**) of *M. aeruginosa* in each treatment group. Notes: The letters indicate the differences between groups on different days, the same letters on the same day indicate that the differences between groups are not significant (*p* > 0.05), and the different letters indicate significant differences (*p* < 0.05).

As demonstrated in Figure 3, the content of Chl a in each treatment decreased from day 4, the value of which was 13.2 μg/L, 6.1 μg/L, and 46.6 μg/L with *E. crassipes*, *P. stratiotes* and *I. aquatica* groups, respectively, on the 15th day. However, the Chl a content in the plastic plant group (the control) showed a significant increasing trend (*p* < 0.05), with a maximum of 216.3 μg/L on day 15 (Figure 3a). *P. stratiotes* showed the highest inhibition on the content of Chl a in *M. aeruginosa*, with inhibition rates up to 97.2%, followed by 93.9% in the presence of *E. crassipes* and 78.5% with *I. aquatica* (Figure 3b). Moreover, by statistically repeated measurements, it was suggested that significant group effects (F = 2834.96, *p* < 0.001), time effects (F = 60.135; *p* < 0.001), and group × time effects (F = 517.82; *p* < 0.001) were detected in the microalgae density of *M. aeruginosa* during the entire experiment.

### 3.3. The Antioxidant Response of Roots, Stems, and Leaves of Different Plants to the Toxic M. aeruginosa

#### 3.3.1. The Activity of CAT

**Figure 4:**
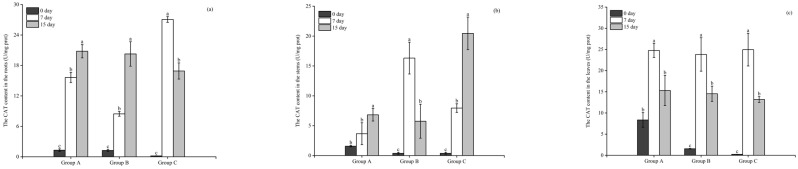
The CAT content in the roots (**a**), stems (**b**), and leaves (**c**) of the three plants. Notes: Different letters in each group indicate the differences in different days, the same letters in each group indicate that the differences in different days are not significant (*p* > 0.05), and different letters indicate significant differences (*p* < 0.05).

The CAT activity in the roots of all three plants was significantly higher (*p* < 0.05) on day 7 than in the initial state. The CAT activities in the roots of *E. crassipes*, *P. stratiotes*, and *I. aquatica* increased to 15.6, 8.46, and 27.06 U/mg prot, respectively, on day 7. At the end of the experimentation on day 15, the CAT activities in the roots of *E. crassipes* and *P. stratiotes* increased significantly to 20.8 and 20.27 U/mg prot, respectively, while that of *I. aquatica* decreased significantly to 16.91 U/mg prot, significantly lower than that of day 7 (Figure 4a).

As illustrated in Figure 4b, the CAT activities in the stems of the *P. stratiotes* and *I. aquatica* increased significantly (*p* < 0.05) to 16.32 and 7.95 U/mg prot, respectively, on day 7. However, no significant increase was detected in the *E. crassipes* group compared with the initial value. On day 15, CAT activity in the stems of *E. crassipes* and *I. aquatica* continued to increase significantly (*p* < 0.05) to 6.82 and 20.46 U/mg prot, respectively, while that of the *P. stratiotes* group decreased significantly to 5.75 U/mg prot compared with the value on day 7.

The CAT activity in the leaves of the three plants varied consistently and increased significantly (*p* < 0.05) from day 0 to day 7, rising to 24.76, 23.82, and 24.95 U/mg prot in the *E. crassipes*, *P. stratiotes*, and *I. aquatica* groups, respectively, and then decreasing to 15.3, 14.54, and 13.18 U/mg prot on day 15 (Figure 4c).

#### 3.3.2. The Activity of GSH

**Figure 5:**
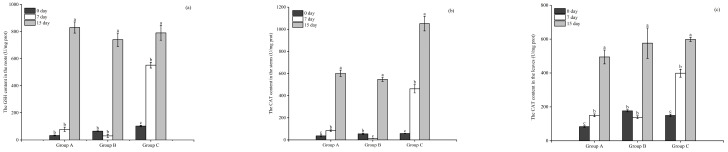
The GSH content in the roots (**a**), stems (**b**), and leaves (**c**) of the three plants. Notes: Different letters in each group indicate the differences in different days, the same letters in each group indicate that the differences in different days are not significant (*p* > 0.05), and different letters indicate significant differences (*p* < 0.05).

Compared with the initial state, the GSH activity in the roots of the three plants showed no significant changes (*p* < 0.05) on day 7 except for the group of *I. aquatica*, the value of which significantly increased to 552.01 U/mg prot on day 7. At the end of the experiment, it was suggested that the activity of GSH in the roots of *E. crassipes*, *P. stratiotes*, and *I. aquatica* were all significantly increased, up to 829.94, 739.99, and 790.36 U/mg prot, respectively (Figure 5a).

On day 7, the GSH activity in the stems of *E. crassipes* and *I. aquatica* were significantly increased to 84.11 and 462.23 U/mg prot, respectively, whereas that of the *P. stratiotes* group was significantly decreased to 12.59 U/mg prot (*p* < 0.05). At the end of the experimentation on day 15, the activities of GSH in the stems of the three plants all significantly increased to 602.34, 547.72, and 1051.27 U/mg prot, respectively (*p* < 0.05) (Figure 5b).

The activities of GSH in the leaves of *E. crassipes* and *I. aquatica* increased significantly to 149.91 and 398.39 U/mg prot, respectively, on day 7 (*p* < 0.05). However, no significant changes were detected in the *P. stratiotes* group compared to the value on day 0. On day 15, the GSH activity in the leaves of the three plants increased to 494.85, 576.27, and 598.35 U/mg prot, respectively, which were all significantly higher than those on days 7 and 0 (*p* < 0.05) (Figure 5c).

#### 3.3.3. The Activity of SOD

**Figure 6:**
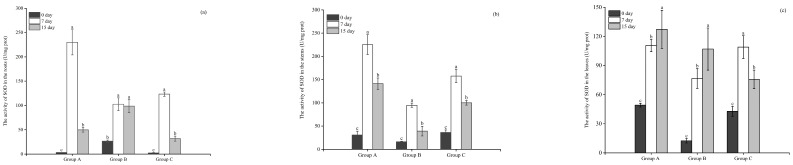
The activity of SOD in the roots (**a**), stems (**b**), and leaves (**c**) of the three plants. Notes: Different letters in each group indicate the differences in different days, the same letters in each group indicate that the differences in different days are not significant (*p* > 0.05), and different letters indicate significant differences (*p* < 0.05).

As illustrated in Figure 6a, the SOD activities in the roots of the three plants were significantly higher on day 7 than on day 0 (*p* < 0.05). The SOD activities in the roots of *E. crassipes*, *P. stratiotes*, and *I. aquatica* were increased to 230.08, 102.67, and 123.38 U/mg prot, respectively, on day 7. No significant changes were detected in the SOD activity of *P. stratiotes* roots between day 7 and 15. The SOD activities in the roots of *E. crassipes* and *I. aquatica* decreased significantly to 49.9 and 31.91 U/mg prot on day 15, which was significantly lower than that on day 7.

The SOD activity in the stems of the three plants changed consistently and increased significantly (*p* < 0.05) from day 0 to day 7, and the value increased to 225.38, 94.8, and 157.71 U/mg prot, respectively, in the stems of *E. crassipes*, *P. stratiotes*, and *I. aquatica* groups, respectively. However, the SOD activity in the stems of the three plants above was decreased significantly (*p* < 0.05) to 141.19, 39.18, and 100.23 U/mg prot, respectively, on the 15th experimental day (Figure 6b).

The SOD activities in the leaves of the three plants increased significantly (*p* < 0.05) during the experimental periods, the value of which in the *E. crassipes*, *P. stratiotes*, and *I. aquatica* groups increased to 110.61, 76.68, and 109.02 U/mg prot, respectively, on day 7, and then continued to increase to 127.35, 107.06 U/mg prot in the leaves of *E. crassipes* and *P. stratiotes* on day 15. However, at the end of the experimentation on day 15, SOD activity in the leaves of *I. aquatica* decreased to 75.76 U/mg prot, which was significantly lower than that on day 7 (Figure 6c).

#### 3.3.4. The Content of MDA

**Figure 7:**
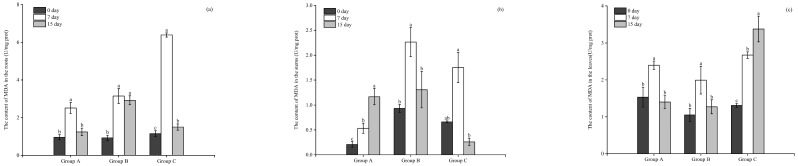
The content of MDA in the roots (**a**), stems (**b**), and leaves (**c**) of the three plants. Notes: Different letters in each group indicate the differences in different days, the same letters in each group indicate that the differences in different days are not significant (*p* > 0.05), and different letters indicate significant differences (*p* < 0.05).

As shown in Figure 7a, the MDA content in the roots of all experimental plants was significantly higher (*p* < 0.05) on day 7 than day 0. The MDA content in the roots of *E. crassipes*, *P. stratiotes* and *I. aquatica* were 2.51, 3.15, and 6.39 nmol/mg prot, respectively, on day 7. No significant changes were detected in the MDA content of the *P. stratiotes* roots between day 7 and day 15, whereas the MDA content in the roots of *E. crassipes* and *I. aquatica* were 1.24, 1.5 nmol/mg prot on day 15, significantly lower than day 7.

The MDA content in the stems of the three plants increased significantly (*p* < 0.05) from day 0 to day 7, the value of which was 0.53, 2.26, and 1.75 nmol/mg prot in the *E. crassipes*, *P. stratiotes*, and *I. aquatica* groups, respectively, on day 7. The MDA content in the stems of *E. crassipes* continued to increase significantly to 1.17 nmol/mg prot on day 15 (*p* < 0.05). However, SOD activity was decreased to 1.31, 0.26 nmol/mg prot in the *P. stratiotes* and *I. aquatica* groups, respectively, which was significantly lower than that on day 7 (Figure 7b).

The MDA content in the leaves of the three plants increased significantly from day 0 to day 7 (*p* < 0.05). The MDA content in the leaves of *E. crassipes*, *P. stratiotes*, and *I. aquatica* were 2.4, 1.99, and 2.67 nmol/mg prot, respectively, and then continued to increase to 3.38 nmol/mg prot in the *I. aquatica* group on day 15, whereas the value of which was significantly decreased to 1.4, 1.27 nmol/mg prot, respectively, in the *E. crassipes* and *P. stratiotes* groups on day 15 (Figure 7c).

### 3.4. Effects of Floating Bed Plants on the MC-LR Content of the Toxic M. aeruginosa

**Figure 8:**
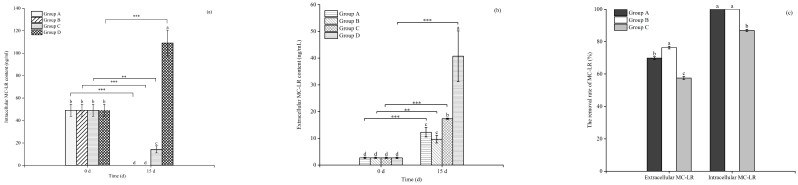
The content of intracellular (**a**), extracellular MC-LR (**b**), and the removal rate of MC-LR by different plants (**c**). Notes (**a**,**b**): Dfferent letters in each group indicate the differences in different days, the same letters in each group indicate that the differences in different days are not significant (*p* > 0.05), and different letters indicate significant differences (*p* < 0.05). ** means *p* < 0.01, *** means *p* <0.001. Notes (**c**): Different letters indicate the differences between different groups, the same letters indicate no significant difference between groups (*p* > 0.05), and the different letters indicate significant difference between groups (*p* < 0.05).

As indicated in Figure 8a,b, the content of intracellular and extracellular MC-LR in the plastic plant (the control group) increased significantly (*p* < 0.05), whereas the content was significantly lower in the treatment with *E. crassipes*, *P. stratiotes*, and *I. aquatica* (*p* < 0.05). The plants *E. crassipes* and *P. stratiotes* showed the best removal effects on the intracellular MCs of *M. aeruginosa*, which could reach up to 100% (Figure 8c).

### 3.5. The Absorbance of MC-LR by the Roots, Stems, and Leaves of the Floating Bed Plants

**Figure 9:**
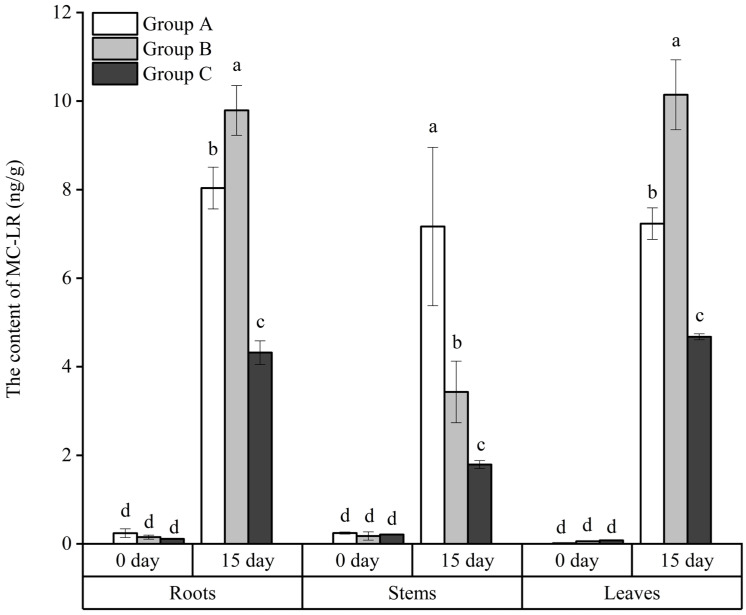
The accumulation of MC-LR by the plant tissues. Notes: Dfferent letters in each group indicate the differences in different days, the same letters in each group indicate that the differences in different days are not significant (*p* > 0.05), and different letters indicate significant differences (*p* <0.05).

The absorbance of MC-LR by the roots, stems, and leaves of the floating bed plants is shown in Figure 9. At the end of the experimentation on day 15, the MC-LR content in the roots, stems, and leaves of *E. crassipes*, *P. stratiotes*, and *I. aquatica* all showed a significant increase (*p* < 0.05), with *P. stratiotes* accumulating the highest MC-LR content in roots and leaves, that was 9.79 and 10.14 ng/g, respectively, followed by *E. crassipes* with 8.04 and 7.23 ng/g, respectively, and *I. aquatica* with 4.32 and 4.68 ng/g, respectively.

### 3.6. Analysis of the Microbial Communities in the Roots of the Plants

**Figure 10:**
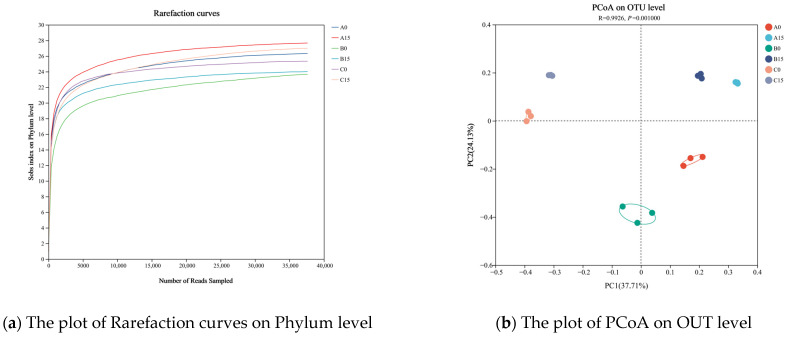
Rarefaction curves and PCoA plot of the microbial community. Note: A0, Group A on day 0; A15, Group A on day 15; B0, Group B on day 0; B1, Group B on day 15; C0, Group C on day 0; C15, Group C on day 15.

The rarefaction curve can directly reflect the rationality of the sequencing data and indirectly reflect the richness of the species in the samples. As can be seen from Figure 10a, with the increase in sample sequencing data, the Sobs index of rhizosphere microorganisms of different plants at the phylum level gradually stabilized, and the diluted curve neared the level, indicating that the sequencing data reflected the vast majority of microbial diversity information in the sample and could be further analyzed.

The species composition of the bacterial communities in each group was analyzed to investigate the similarity or difference in the bacterial community composition of different plants from day 0 to day 15 of the experiment. The Bray–Curtis distance algorithm was used at the OUT level, and PCoA analysis was used to analyze the differences in the root microbial communities of the plants. The results are shown in Figure 10b. The PC1 and PC2 of the plant root microbial communities explained 37.71% and 24.13% of the variance, respectively, with a cumulative explanation ability of 61.84%. There were significant differences among the microbial communities of the groups (ANOSIM, R = 0.9926, *p* = 0.001000), and there was a clear separation of the microbial communities of the three floating bed plants from day 0 to day 15. The three replicate samples from each group showed good clustering, indicating good repeatability, which suggests that the bacterial community composition within each group was relatively small.

**Figure 11:**
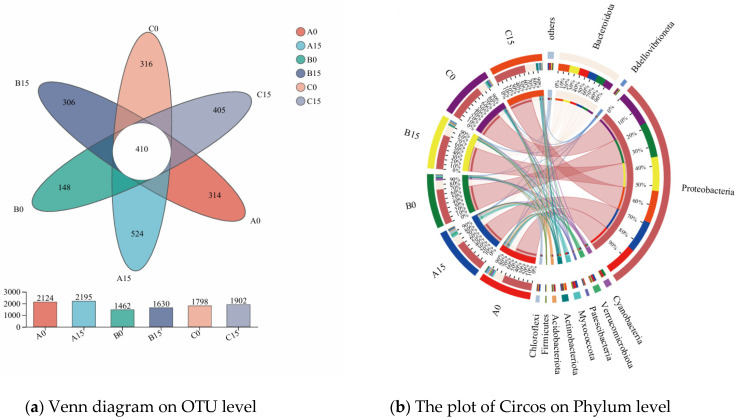
Venn diagram and Circos plot of the microbial community. Note: A0, Group A on day 0; A15, Group A on day 15; B0, Group B on day 0; B1, Group B on day 15; C0, Group C on day 0; C15, Group C on day 15.

Venn plots show the distribution of operational taxonomic unit (OTU) numbers in the biofilms of the plants and provide a visual visualization of the number of common and unique OTUs, showing compositional similarities and overlaps across samples. As illustrated in Figure 11a, a total of 410 OUTs were detected in the six treatment groups, with the highest amount of OUTs in the group of *M. aeruginosa* with *E. crassipes* on day 15 and the lowest amount in the group of *M. aeruginosa* with *P. stratiotes* on day 0. Meanwhile, it was also suggested that the OUTs numbers were markedly higher on day 15 than on day 0 in all treatment groups.

Circos plots are usually used to show the distribution of microbial species present in different microbial samples (Figure 11b), in which *Proteobacteria* and *Bacteroidota* were dominant in all six groups, accounting for 15–18% and 14–19%, respectively, and *Cyanobacteria* accounted for 28%, 46%, and 9% of the total number of samples in the group of *M. aeruginosa* with *E. crassipes*, *M. aeruginosa* with *P. stratiotes*, and *M. aeruginosa* with *I. aquatica*, respectively, on day 0, whereas on day 15, the value of which dropped to 5%, 4%, and 8% on day 15. *Verrucomicrobiota* and *Myxococcota* were higher in the group of *M. aeruginosa* with *E. crassipes* on day 0, accounting for 22% and 29%, respectively, and on day 15, accounting for 30% and 33%, respectively.

**Figure 12:**
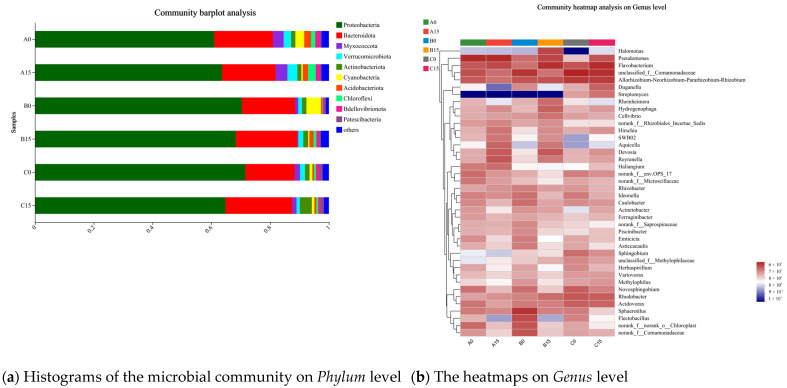
Histograms of the microbial community at the *Phylum* level and heatmaps at the *Genus* level for the different treatment groups. Note: A0, Group A on day 0; A15, Group A on day 15; B0, Group B on day 0; B1, Group B on day 15; C0, Group C on day 0; C15, Group C on day 15.

The microbial community at the phylum level is shown in Figure 12a, and it was indicated that the main dominant phylum was *Proteobacteria*, accounting for 60.97–71.57%, and *Bacteroidota*, accounting for 16.81~22.81%, in the three plant treatment groups. *Myxococcota* and *Verrucomicrobiota* had the highest occupancy of 3.9% and 3.4% in the group of *M. aeruginosa* with *E. crassipes* on day 15, and *Actinobacteriota* had the highest occupancy of 4.1% in the group of *M. aeruginosa* with *I. aquatica* on day 15. *Cyanobacteria* were higher in the group of *M. aeruginosa* with *E. crassipes* and *M. aeruginosa* with *P. stratiotes* on day 0, accounting for 3% and 4.9%, respectively, and decreased significantly to 0.51% and 0.41% on day 15, while *Chloroflexi* was significantly higher on day 15 than that of day 0 in the group of *M. aeruginosa* with *E. crassipes* and *M. aeruginosa* with *P. stratiotes*.

The heatmap in Figure 12b demonstrates the differences in microbial composition at the genus level in each treatment group between the start and end of the experiment. A total of the top 20 genera were identified, in which *Allorhizobium-neorhizobium-pararhizobium-rhizobium* and *Flavobacterium* were dominant in all treatment groups, and their amounts were significantly higher than those of other genera. In addition, the amount of *Aquicella* spp. was significantly higher in the groups of *M. aeruginosa* with *E. crassipes* and *M. aeruginosa* with *P. stratiotes* on day 15. The amount of *Flectobacillus* was significantly lower in the group of *M. aeruginosa* with *E. crassipes*, *M. aeruginosa* with *P. stratiotes* and *M. aeruginosa* with *I. aquatica* on day 15 than in the initial state. The abundance of *Halomonas* was significantly higher in the group of *M. aeruginosa* with *P. stratiotes* on the 15th day than that in the other groups. *Duganella* was significantly higher in the group of *M. aeruginosa* with *E. crassipes* and *M. aeruginosa* with *P. stratiotes* on day 15 than that of day 0, but higher in the group of *M. aeruginosa* with *I. aquatica* on day 15 than that in the group of *M. aeruginosa* with *I. aquatica* on day 0. *Streptomyces* was significantly lower in the groups of *M. aeruginosa* with *E. crassipes* and *M. aeruginosa* with *P. stratiotes* than in the group of *M. aeruginosa* with *I. aquatica*. *Rheinheimera* showed a decreasing tendency from day 0 to day 15 in the group of *M. aeruginosa* with *E. crassipes* and *M. aeruginosa* with *I. aquatica*, but demonstrated an increasing tendency in the group of *M. aeruginosa* with *P. stratiotes*.

## 4. Discussion

### 4.1. Microalgae Suppression Effects on M. aeruginosa by Floating Bed Plants

Allelopathy is considered one of the most important ecological mechanisms for the control of *M. aeruginosa* growth, which has been widely demonstrated in previous studies [27]. In the present study, we found that all three floating bed plants had significant inhibitory effects on the growth of *M. aeruginosa* during the experimental period, with *P. stratiotes* having the greatest inhibitory effects on the growth of *M. aeruginosa*. Reynolds (2021) [28] found that allelochemicals produced by aquatic plants during growth can be transferred to the roots and secreted into water to inhibit phytoplankton growth. In this experiment, the roots of floating bed plants were in direct contact with *M. aeruginosa*, further validating the plant’s anti-algalescence effect. A study by Pei et al. (2018) [29] found that *E. crassipes* was effective in inhibiting the growth of *Cyanobacteria*, and Cheng et al. (2021) [30] also showed that *E. crassipes* had a great inhibitory effect on the growth of *Cyanobacteria* because of its enormous root biomass and allelopathic substances (including phenylnaphthylamines, linolein derivatives, and phenalenes). Zhou et al. (2019) [31] indicated that *Cyperus alternifolius* and *Canna generalis* contain a total of 15 kinds of compounds, including fatty acids and phenolic compounds, which are anti-cyanobacterial. Nakai et al. (2010) [32] showed that *Typha angustifolia*, *Scirpus tabernaemontani*, and *Phragmites australis* release anti-cyanobacterial compounds from their roots into the culture solution, which inhibits cyanobacterial growth. Moreover, Van Nguyen et al. (2019) [33] indicated that the extracts of *E. crassipes* and *P. stratiotes* could effectively inhibit the growth of *M. aeruginosa* and could be used as algaecides to control *M. aeruginosa* blooms. Wijewickrama and Manage (2019) [24] also proved the inhibitory effects of *I. aquatica* on the growth of *Microcystis.* These findings provide a basis for the application of floating-bed plants for cyanobacterial inhibition via allelopathic effects.

### 4.2. Antioxidant Response of the Floating-Bed Plants to Toxic M. aeruginosa

MDA is the final product of membrane lipid peroxidation and directly indicates the extent of damage to the membrane systems [34]. CAT and G-Px are protective enzymes in the plant antioxidant system. When hydrogen peroxide levels increase in plant cells, the expression of CAT also increases correspondingly [35]. G-Px plays a crucial role in protecting cells from oxidative damage within plants [36], with selenium as its cofactor, which facilitates the decomposition of peroxides [37] and plays a major role in ROS defense mechanisms [38]. SOD is a metalloenzyme whose distribution varies among different organisms and is primarily localized in mitochondrial organelles. Plants respond to stress when external environmental conditions change [39]. In the present study, the three kinds of floating bed plants showed different responses to toxic *M. aeruginosa*. The results showed that the experimental plants were stressed by external environmental factors (e.g., high nutrient salts, MCs) and protected themselves from damage by their own antioxidant systems, thus, reducing damage to the cell membrane structure. Previous studies have suggested that the antioxidant capability of plants is closely related to the development of their associated stress tolerance ability [40]. Chen et al. (2004) [41] showed that exposure to microcystins inhibited the growth and development of both rice and rape seedlings, and the activities of POD and SOD demonstrated that microcystin stress was manifested as oxidative stress. Ge J (2012) [42] studied the antioxidant responses of the macrophyte *Vallisnerria natans* seed-lings to microcystin-LR (MC-LR) for 7 days. The results showed that SOD, CAT, POD, and GSH levels were significantly induced by MC-LR. Previous research has illustrated that the activities of these enzymes increased following exposure to MCs, protecting plants from oxidative damage induced by MCs, such as POD and CAT in spinach [43]. Wang and Wang (2018) [44] also found that CAT, POD, and SOD contents in *Iris pseudacorus* were significantly increased by MC-LR stress.

### 4.3. The MCs Removal and Microbial Response of the Plants to MCs

The results of the study showed that the content of the extracellular toxin in all experimental groups showed a significant increase, which might be due to the release of toxins from the decay of *M. aeruginosa*, leading to an increase in extracellular toxin. Jones & Orr (1994) [45] have confirmed that more MCs can be released during the decay and degradation processes of toxin-producing *M. aeruginosa*. However, compared to the increase in MC-LR detected in the control group, the toxin content was greatly reduced in the treatment group. In particular, intracellular toxin content in the *M. aeruginosa* group with *E. crassipes* or *P. stratiotes* was not detected. Furthermore, floating bed plants were able to adsorb and decompose MCs in the water, which helped to reduce toxin levels in the water column. At the end of the experiment, it was found that the MCs were distributed in the plant tissues, as evidenced by a significant increase in the MC-LR content of the plant tissues (*p* < 0.05). Pflugmacher et al. (2001) [46] also found that *Phragmites australis* could rapidly take up MC-LR and was clearly distributed in different parts of the plants. A study by Saqraneet et al. (2007) [47] also demonstrated MCs accumulation, detoxification, and oxidative stress induction in *Lemnaceae* tissues, revealing the uptake and biotransformation of MCs by floating plants.

Microbial membranes play an important role in energy flow and nutrient cycling [26]. In this study, 1542 species belonging to 35 phyla, 95 orders, 238 phyla, 401 families, and 779 genera were detected in the microbial communities in the root systems of the plants. The percentage of *Cyanobacteria* decreased, and the percentage of *Chloroflexi* increased in the group of *M. aeruginosa* with *E. crassipes* or *P. stratiotes*. The dominant species in each treatment group were mainly *Proteobacteria* and *Bacteroidota*, which are common freshwater taxa and are also dominant in microbiological studies of ecological floating bed systems [48,49]. Reciprocal symbiosis exists between bacteria and plants [50]. Some bacteria belonging to the phylum *Proteobacteria* have the ability to fix nitrogen, and they can convert nitrogen in the air into a source of nitrogen that is available to plants, thus providing them with important nutrients [51]. *Bacteroidota* are specialized anaerobic bacteria. Some bacteria in *Bacteroidota* can produce antibiotics, which can inhibit the growth of pathogenic bacteria in water bodies, and play a biological defense role for plants, which can help improve plants’ resistance to diseases [52]. Liu et al. (2023) [53] found that the dominant microbial phyla in the root microorganisms of rice were *Proteobacteria* and *Bacteroidota*. Zhang et al. (2022) [54] also showed that *Proteobacteria* and *Bacteroidota* are the dominant phyla of root microorganisms in aquatic plants in ecological floating beds. Studies have indicated that bacteria can target cyanobacteria through mechanisms such as attachment, allelochemical release, or a combination of both strategies [33]. Additionally, relevant literature suggests that certain bacteria can inhibit the growth of cyanobacteria by producing organic compounds that suppress their proliferation [55]. It has also been found that, under the stress of cyanobacterial toxins, these two dominant species are usually present in the microbial membranes attached to plants [17], maintaining the stability of the biofilm function [56]. Lezcano MÁ et al. (2017) [57] suggests an important role played by the mlr-lacking bacteria for the degradation of MCs and/or other cyanobacterial exudates. Most of these bacteria, particularly *Proteobacteria* and *Bacteroidetes*, have been reported to degrade complex organic compounds [58]. Gao et al. (2023) [59] showed that *Proteobacteria* and *Bacteroidetes* contain strains with algicidal and MC-degrading capabilities.

Furthermore, Some studies have found that a variety of microorganisms can degrade MCs, providing an important biological tool for controlling the MC pollution [60]. Scherer et al. (2017) [61] found that there are potential microcystin-degrading bacteria and potential algaecicidal bacteria among the ubiquitous *Bacteroidetes* and *Alphaproteobacteria*. Zhu et al. (2016) [62] found that degrading cyanobacterial toxins revealed that these bacteria belong primarily to the phylum *Proteobacteria*, including several strains of *Sphingomonas* and two strains belonging to the *Methylobacillus* and *Paucibacter*, respectively. Bourne et al. (2006) [63] suggested that the MC degradation process is an interaction between different bacterial communities and MC-degrading bacteria immobilized in biofilms. A variety of microorganisms can also be involved in the degradation of MCs content [64]. A study by Zeng et al. (2020) [65] found that *Phanero-chaetechrysosporium* not only inhibited the growth of *M. aeruginosa*, but its metabolites were also able to inhibit the synthesis of MCs and significantly block the metabolic system of *M. aeruginosa* growth and microcystin synthesis. Salter et al. (2021) [66] also indicated that bacterial communities are very effective in degrading MC-LR. Jones et al. (1994) [67] reported that bacteria such as *Pseudomonas* were effective in degrading MCs, and in the present study, *Pseudomonas* was found to be widespread in each of the experimental groups. He et al. (2022) [68] suggested that MC-degrading bacteria include *Sphingomonas*, *Acinetobacter*, *Arthrobacter*, *Bacillus*, *Novosphingobium*, *Pauci-bacter*, *Pseudomonas*, *Sphingopyxis*, and *Stenotrophomona*. In the present study, *Sphingo-monas*, *Acinetobacter*, *Novosphingobium*, and *Pseudomonas* were also present in large amounts in all experimental groups, verifying the hypothesis that microorganisms are involved in the degradation of MCs. Dziga et al. (2019) [69] found that the microbial degradation of MCs can effectively reduce the concentration of toxins in water, protect the health of aquatic ecosystems, and maintain biological diversity. Microbial degradation of MCs can also reduce toxins in drinking water sources and human health risks from water pollution [70] (Massey IY et al. 2020).

### 4.4. Conclusions and Prospect

All three types of floating plants had an inhibitory effect on the growth of *M. aeruginosa*, with *E. crassipes* being the most effective, followed by *P. stratiotes* and *I. aquatica*. In addition, all three plants demonstrated good MCs accumulation, and *E. crassipes* and *P. stratiotes* had the best MC removal effects. Toxin-degrading bacteria were also detected in the present study, providing important basic data for controlling MCs pollution. However, during exposure to toxic *M. aeruginosa*, oxidative responses were also detected, as evidenced by the significant increases in the levels of CAT, MDA, GSH-PX, and SOD in the roots, stems, and leaves of the floating bed plants.

The research system is only 5 L; therefore, it is necessary to carry out large-scale or in situ water simulation experiments in the future to provide further scientific reference for the restoration of cyanobacterial blooms in bloom water. Microbial communities have an important impact on the ecological function of water bodies; however, the impact of ecological floating beds on microbial diversity, structure, and function is currently poorly understood. In addition, research on the removal of *Microcystins* by ecological floating beds remains in the preliminary stage. Although some studies have shown that floating bed plants can reduce the concentration of microcystins in water bodies, an in-depth understanding of their removal mechanisms and effects remains insufficient. To better understand its role, further research is needed to explore in depth its impact mechanisms on microbiome community dynamics, the effect of microcystella toxin removal, and to optimize its design and operational strategies to achieve more sustainable water environment management and protection.

## Data Availability

Data are contained within the article. All sequence data have been deposited in the NCBI sequence read archive at accession number PRJNA1164027.
